# Rare variants and HLA haplotypes associated in patients with neuromyelitis optica spectrum disorders

**DOI:** 10.3389/fimmu.2022.900605

**Published:** 2022-10-04

**Authors:** Inna Tabansky, Akemi J. Tanaka, Jiayao Wang, Guanglan Zhang, Irena Dujmovic, Simone Mader, Venkatesh Jeganathan, Tracey DeAngelis, Michael Funaro, Asaff Harel, Mark Messina, Maya Shabbir, Vishaan Nursey, William DeGouvia, Micheline Laurent, Karen Blitz, Peter Jindra, Mark Gudesblatt, Regeneron Genetics Center, Alejandra King, Jelena Drulovic, Edmond Yunis, Vladimir Brusic, Yufeng Shen, Derin B. Keskin, Souhel Najjar, Joel N. H. Stern

**Affiliations:** ^1^Department of Neurology, Donald and Barbra Zucker School of Medicine at Hofstra/Northwell, Hempstead, NY, United States; ^2^Department of Urology, Donald and Barbra Zucker School of Medicine at Hofstra/Northwell, Hempstead, NY, United States; ^3^Department of Molecular Medicine, Donald and Barbra Zucker School of Medicine at Hofstra/Northwell, Hempstead, NY, United States; ^4^Department of Science Education, Donald and Barbra Zucker School of Medicine at Hofstra/Northwell, Hempstead, NY, United States; ^5^Institute of Molecular Medicine, The Feinstein Institutes for Medical Research, Manhasset, NY, United States; ^6^Department of Neurobiology and Behavior, The Rockefeller University, New York, NY, United States; ^7^Department of Pediatrics, Columbia University College of Physicians and Surgeons, New York, NY, United States; ^8^Department of Biomedical Informatics and Department of Systems Biology, Columbia University, New York, NY, United States; ^9^Department of Computer Science, Boston University, Boston, MA, United States; ^10^Clinical Center of Serbia University School of Medicine, Belgrade, Serbia; ^11^Department of Neurology, University of North Carolina School of Medicine, Chapel Hill, NC, United States; ^12^Biomedical Center and University Hospitals, Ludwig Maximilian University Munich, Munich, Germany; ^13^Department of Neurology, Neurological Associates of Long Island, New Hyde Park, NY, United States; ^14^Department of Neurology, Lenox Hill Hospital, Northwell Health, New York, NY, United States; ^15^Department of Neurology, South Shore Neurologic Associates, Patchogue, NY, United States; ^16^Division of Abdominal Transplantation, Baylor College of Medicine, Baylor College of Medicine, Houston, TX, United States; ^17^Regeneron Genetics Center, Regeneron Pharmaceuticals Inc., Tarrytown, NY, United States; ^18^Department of Medical Oncology, Dana-Farber Cancer Institute, Boston, MA, United States; ^19^School of Computer Science, University of Nottingham Ningbo China, Ningbo, China; ^20^Department of Translational Immuno-Genomics for Broad Institute of Massachusetts Institute of Technology and Harvard, Cambridge, MA, United States; ^21^Department of Medicine, Brigham and Women’s Hospital, Boston, MA, United States

**Keywords:** neuromyelitis optica spectrum disorders, human leukocyte antigen, exome sequencing, autoimmunity, antibody mediated

## Abstract

Neuromyelitis optica spectrum disorders (NMOSD) are rare, debilitating autoimmune diseases of the central nervous system. Many NMOSD patients have antibodies to Aquaporin-4 (AQP4). Prior studies show associations of NMOSD with individual Human Leukocyte Antigen (HLA) alleles and with mutations in the complement pathway and potassium channels. HLA allele associations with NMOSD are inconsistent between populations, suggesting complex relationships between the identified alleles and risk of disease. We used a retrospective case-control approach to identify contributing genetic variants in patients who met the diagnostic criteria for NMOSD and their unaffected family members. Potentially deleterious variants identified in NMOSD patients were compared to members of their families who do not have the disease and to existing databases of human genetic variation. HLA sequences from patients from Belgrade, Serbia, were compared to the frequency of HLA haplotypes in the general population in Belgrade. We analyzed exome sequencing on 40 NMOSD patients and identified rare inherited variants in the complement pathway and potassium channel genes. Haplotype analysis further detected two haplotypes, HLA-A*01, B*08, DRB1*03 and HLA-A*01, B*08, C*07, DRB1*03, DQB1*02, which were more prevalent in NMOSD patients than in unaffected individuals. *In silico* modeling indicates that HLA molecules within these haplotypes are predicted to bind AQP4 at several sites, potentially contributing to the development of autoimmunity. Our results point to possible autoimmune and neurodegenerative mechanisms that cause NMOSD, and can be used to investigate potential NMOSD drug targets.

## Introduction

Neuromyelitis optica spectrum disorders (NMOSD) are inflammatory disorders of the central nervous system marked by astrocyte damage, severe immune-mediated demyelination and axonal damage of the optic nerves and the spinal cord (1, 2). NMOSD has an estimated prevalence between 0.5 and 10 cases per 100,000 in the population (3–5), but patients may still be underdiagnosed due to the similarity of symptoms to other autoimmune neurodegenerative diseases, such as multiple sclerosis (MS), and the expanding spectrum of humorally mediated autoimmune syndromes of the CNS in NMOSD (6). The clinical distinction between NMOSD and MS, particularly in early diseases stages, may be difficult because of a considerable overlap between the symptoms; however, accurate diagnosis is important as patients with NMOSD require different treatment (2, 7).

NMOSD was first described in a series of patients in 1894 (8), but a unique biomarker was not identified until 2004, when a disease-specific serum NMOSD-immunoglobulin G (IgG) antibody was detected in the vast majority of patients with NMOSD (9–12). It was not observed in individuals with other autoimmune neurodegenerative diseases (9–12). The NMOSD IgG antibody binds to astrocyte water channel protein Aquaporin-4 (AQP4): a member of the aquaporin water-selective membrane channel family of proteins. In humans, AQP4 is the predominant water channel protein in the brain. It is prevalent in astrocytes at the blood-brain barrier and has an essential role in brain water homeostasis (13). It has been demonstrated in several models that AQP4 autoantibodies have a pathogenic role in NMSOD, and that they lead to tissue destruction primarily through complement-mediated cytotoxicity (13–18). NMOSD is characterized by astrocytic cell death and destruction of the blood-brain-barrier (BBB), while MS primarily affects oligodendrocytes (16, 19, 20). Destruction of the BBB in NMOSD may partially be mediated by AQP4-IgG antibodies (21) and by pathogenic autoreactive T cells (22–25). Neuronal cell death and oligodendrocyte damage are considered as secondary events (26).

In a subgroup of NMOSD patients with a diverse clinical phenotype, autoantibodies to a myelin specific glycoprotein (MOG) can be found at high titers (27–30). This group of patients is most recently defined as MOG associated diseases (MOGAD) (31, 32). It is becoming increasingly clear that patients with AQP4 IgG positive NMOSD and those with MOGAD manifest two separate CNS demyelinating diseases with different biological, clinical, and neuropathological characteristics. NMOSD most commonly presents as acute attacks of bilateral or rapidly sequential optic neuritis, or characteristic MRI findings of long spanning spinal cord lesions and/or brain lesions. Patients with variable presentation are also classified as having NMOSD (8). Patients with AQP4 IgG antibodies detected in blood have been associated with NMOSD whereas the presence of MOG antibodies leads to a diagnosis MOGAD (29).

NMOSD predominantly occurs in adults and is tenfold more prevalent in women than in men (33–35). Women are more frequently affected by relapsing disease (35). The average age of NMOSD onset is between 32 and 41 years of age, but it can be quite variable (36, 37), while the onset of MS commonly in the early twenties. Based on limited population data, AQP4 seropositive NMOSD is more prevalent in populations of African and Afro-Caribbean descent in the US (5), while rates of MS are thought to be higher among people of European ancestry (38).

Previous studies have described an association with NMOSD and different alleles of the Human Leukocyte Antigen (HLA), which are variable between populations (39). Studies of genetic susceptibility of NMOSD have indicated an association with HLA DRB1*03:01 in French, Brazilian, Mestizo Mexican and Afro-Caribbean populations, but not in Danish, Spanish, or Arab populations (40–45). Some of these patients were seronegative for AQP4 antibodies, but still met the criteria for NMOSD. In Japan, NMOSD was associated with HLA-DRB1*1602 and DPB1*0501 alleles (46). Genetic associations of NMOSD with alleles outside the HLA region are only starting to be explored. Genome wide association studies (GWAS) of NMOSD (43–46) have shown an association with single nucleotide polymorphisms (SNPs) located near genes encoding complement proteins and several candidate genes involved in NMOSD, including a potassium channel gene, KCMA1 (47–49).

In our present study we performed HLA haplotype analysis in addition to whole exome sequencing of individuals with NMOSD to identify candidate genes and pathways that underlie the pathogenesis of NMOSD disease.

NMOSD rarely affects multiple members of the same family. This could be due to environmental or other factors, including *de novo* and somatic mutations, which have recently been associated with multigenic inheritance in patients with neurodevelopmental and psychiatric conditions (50, 51). To understand whether *de novo* mutations could contribute to NMOSD, and to identify candidate genes and pathways, we used trio analysis of exome sequencing data in families in which at least one member was affected by NMOSD.

## Material and Methods

### Patients

The present study was approved by the Northwell Health Institutional Review Board. NMOSD patients (first and second degree) who met the revised diagnostic criteria for NMOSD were enrolled at the Clinic of Neurology Clinical Center of Serbia (n=26, referred to as Serbian patients) and at Northwell Health and Lenox Hill Hospital, Neurological Associates of Long Island, and South Shore Neurologic Associates, New York, USA (n=14). In total, 33 females and seven male patients were enrolled. Only NMOSD patients without any clinical evidence of other autoimmune diseases were included in this study.

Blood samples were collected from patients affected with NMOSD according to protocols approved by the Northwell Health Institutional Review Board (IRB# HS15-06-03). Written informed consent was obtained from all participants.

### Cell-based antibody assay

All NMOSD patients and relatives were screened for antibodies against AQP4 (M23 isoform) and human MOG using a cell-based assay as previously described (30, 52) Samples were tested using HEK 293T cells transfected with relevant epitopes, as well as untransfected negative controls. HEK 293T cells were used 24 hours after transfection with MOG, or 72 hours after transfection with AQP4. To test for antibody binding we first blocked with PBS/10% fetal calf serum (FCS) for an hour, then incubated with patient serum diluted with PBS/10% FCS at 4°C for an hour.To visualize antibody staining, cells were incubated with Alexa-594 conjugated goat anti-human IgG (Life Technologies) for 30 minutes at room temperature, then washed in PBS/10% FCS, stained with DAPI to assess the viability and visualized with a fluorescent microscope. Titer ≥ 1:160 were classified as MOG-IgG positive (30).

### High Resolution HLA typing

HLA typing was performed with genomic high resolution typing (second field) of HLA- A, B, C, DRB1, DRB3, DRB4, DRB5, DQA, DQB, DPA and DPB. All loci were analyzed by PCR-sequence specific oligonucleotide (PCR-SSO) probing using LABType SSO HD kits (One Lambda). A Luminex 200 (Luminex Corporation) flow analyzer was used to identify the fluorescence intensity of PE (phycoerythrin) of PCR products bound to fluorescently coded microspheres. The data were imported into HLA Fusion software version 4.4.0 (One Lambda) for analysis.

### Statistics

#### Analysis of HLA sequencing

Two datasets of HLA allele frequencies in Serbian populations from Allele Frequency Net Database (52) were used to compare HLA profiles of NMOSD patients to the general Serbian population. The dataset named “Serbia_pop3” was derived from HLA typing of 1,992 volunteer bone marrow donors (53). The dataset named “Serbia_Vojvodina” was derived from HLA typing of a sample population of 400 Serbians. Dataset Serbia_pop3 was used for comparison to the patient population, as it was based on a much larger sample. Our HLA typing is of higher resolution than that of Serbia_pop3, which is in 2-digit resolution. To make the results comparable, we computationally reduced our typing result to two decimal places, corresponding to nonsynonymous substitutions only (**Supplementary Table 5**). Using the Fisher eact test, we compared allele frequency in our Serbian patient cohort with the general Serbian population. The investigators were blinded to the clinical information of the patients in the analysis.

### Peptide binding predictions

NetMHC 4.0 (54, 55) and NetMHCII 2.2 servers (DTU Health Tech, Lyngby, Denmark) (56) were used for HLA class I and class II binding predictions, respectively. For class I binding prediction, thresholds used for strong and weak binders were top 1% rank and top 2% rank respectively. For class II binding prediction, thresholds used to define strong and weak binders were affinities of ≤ 50nM and ≤500nM, respectively.

### Visualization of HLA binding to AQP4

Pymol software (Schrodinger, Inc.) was used to model the protein structure of AQP4 (3GD8). The membrane spanning helices were identified based on the existing literature (22). Peptides were identified with the software based on the sequence. The intracellular region of AQP4 was identified based on the presence of N and C terminals. The peptide sequences closer to the N and C terminals could not be visualized because they had not been included in the crystal structure.

### Whole exome sequencing analysis

**Figure 1:**
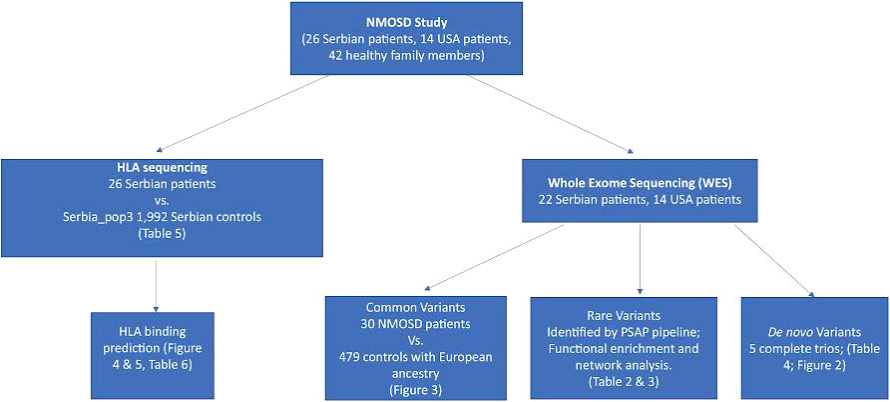
Analysis workflow. Our datasets were obtained from 26 Serbian patients and 14 US patients. For Serbian patients, we performed HLA sequencing and compared allele frequencies to 1,992 controls with the same ancestry. We then predicted the binding affinity of HLA proteins encoded by the alleles associated with NMOSD to AQP4 and MOG. Candidate mutations associated with NMOSD were identified in 36 patients through whole exome sequencing. Common, rare and *de novo* (when DNA from family members was available) potentially deleterious variants were identified.

Whole exome sequencing was performed on 36 individuals, including 5 trios (**Figure 1**), 21 singletons, and 10 families with first and/or second degree relatives. Clinical characteristics of the patients are listed in **Table 1**. (Detailed patients information listed in **Supplementary Table 4**). Overall, 33 of 36 NMOSD patients where AQP4 IgG positive and none were MOG IgG positive. (**Table 1**). All relatives were tested negative for AQP4 IgG and MOG IgG. DNA for whole-exome sequencing was isolated using Qiagen DNA isolation kit. Whole exome sequencing was performed at Regeneron Genetics Center (Tarrytown, NY, USA). Exome sequence was captured by Xgen kit (Integrated DNA Technologies, Inc., Coralville, IA, USA) and sequenced on an Illumina Hi-seq 2500 (Illumina) with 78 base pair paired-end reads and read-depth coverage of at least 15X for 95% of target regions for all samples.

**Table 1:** Clinical and demographic characteristics of NMOSD Patients.

	NMOSD patients
	(n = 40)
Female (%)	80.8
Mean Age	47.6 (std=11.6)
AQP4-IgG Positive (%)	92.3
Clinical Presentations (%):	
Optical neuritis (ON)	65.4
Visual Loss	65.4
Myelopathy	92.3
Motor Weakness	92.3

Data were processed by a bioinformatics pipeline based on Genome Analysis Toolkit (GATK) best practice. Reads were mapped to HG19 by BWA-mem algorithm (57). GATK-haplotype caller and GenotypeGVCF were used for variant discovery and joint genotyping the whole cohort. SmartPCA and in-house script based on a random forest algorithm were used for determining the populations of patients (58, 59). ANNOVAR was used to annotate the consequence of variants and aggregate information about allele frequencies and *in silico* prediction algorithms of deleteriousness prediction (57).

All variants were filtered for Genome Aggregation Database (gnomAD) and ExAC allele frequency < 0.01, Genotype quality (GQ) > 60, minimal read depth of 5, minimal allele fraction of 0.1, Fisher allele balance test score(FS) < 25, Quilyt per depth(QD) > 2, segmental duplications < 0.95 and mappability >= 1. which are more stringent than common variants, since rare variants usually have a higher false positive rate than common variants. We used Population Sampling Probability (PSAP) to identify potential rare variants as disease candidates (59) to address the small size of our cohort limiting the power of a case-control burden test. We ran PSAP on all patients, filtered for rare variants as Minor Allele Frequency (MAF) <0.1%, and assess the p-value of how likely this variant was to be seen in the general population (ExAC) (60). More specifically, we used the following parameters: popscore < 1e-3, MAF in gnomAD and ExAC < 1e-3, Combined Annotation Dependent Depletion (CADD) score >= 20 to identify genes most likely to contribute to NMOSD. Variants were classified according to the ACMG variant interpretation guidelines (61). In addition to adhering to the ACMG criteria, our analysis specifically focused on variants that occurred in a gene with a known function, were shared among all affected individuals in our cohort, and predicted as deleterious by multiple *in silico* prediction algorithms including Combined Annotation Dependent Depletion (CADD) (62) and Rare Exome Variant Ensemble Learner (REVEL) (63). Functional enrichment of gene harbouring damaging mutations were performed with Enrichr (64). To identify whether these genes have interactions with each other to achieve same biological functions, we performed protein-protein interaction (PPI) network analysis with STRING database (https://string-db.org/, v10.5), and corresponding network were constructed using candidate genes selected by PSAP. Genes are represented by nodes and interaction between genes are represented by edges. Types of interactions were restricted based on experimental evidence, interaction databases and co-expression. The minimum requirement of evidence score was set to 0.9, which resulted in only the highest confidence of interaction.

#### Common SNP association

To determine whether an association exists between NMOSD and common SNPs, we selected common variants (MAF > 1%) identified in the individuals. Control data were obtained from other disease cohorts, which were sequenced together with our NMOSD patients in the same sequencing batch. These cohorts are case-control studies of other diseases such as breast cancer, cardiomyopathy and obesity. We selected healthy family members from these cohorts as the controls, and potential technical batch effects that may impact our GWAS study were thus minimized. Genome-wide Complex Trait Analysis (GCTA) (65) was used to check pedigrees and select index patients from control data. European cases and controls were used to perform association tests to avoid population stratification. Eigensoft (66) was used to perform principal component analysis (PCA) and an in house script based on random forest algorithm was used to select European individuals according to 1KG references. After selecting on population and relatedness, 30 NMOSD cases and 479 controls with European ancestry were selected. Wald test for logistic regression (b.wald) test implemented in Efficient and Parallelizable Association Container Toolbox (EPACTS) was used to perform statistical testing on selected SNPs and individuals. The first 10 eigen values from population PCA were used as covariates. For variant quality, filters on GQ > 30 and allele fraction in [0.3, 0.7] were applied. Variants that failed these filters were marked and not called.

## Results

### Identification of rare, deleterious variants by whole exome sequencing

Analysis of Whole Exome Sequencing (WES) data identified several rare variants (MAF < 1% of the general population) in genes potentially associated with NMOSD. Rare variants are increasingly becoming understood to play an important role in disease susceptibility to multiple disorders, including autoimmune neurodegenerative diseases, such as multiple sclerosis (MS) (67). After applying the filters for high-confidence rare-variant screening (see Method), we observed 346 statistically significant variants in 338 genes. The majority of the alleles were heterozygous missense variants (**Supplementary Table 1**). Two immune related pathways, Class I MHC mediated antigen processing and MHC class II antigen (**Supplementary Table 2**), were identified to be enriched with selected rare damaging missense variants. Mutations in a potassium channel have been previously proposed to confer susceptibility to NMOSD (49), and mutations in potassium channels were also identified to be enriched with rare damaging missense variants in our patient cohort (68). The PPI network analysis gave us three groups of highly interconnected genes with predicted damaging mutations in our exome sequencing patient cohort. The first set of genes, including UBE2Q1, HUWE1, UBE2H, ASB8, ZNRF1 and TRIM37, are pairwise connected in network and form the function of class I MHC mediated antigen processing. The second gene group consisted of MHC class II antigen genes: DCTN1, CANX, DCTN3 and OSBPL1A. The third network showed DCTN1, CLTC, DRD4, OSBPL1A and CCR4, which are genes involved in neuroactive ligand-receptor interaction, G alpha (i) signaling events and G Protein Coupled Receptors (GPCRs)downstream signaling.

### Rare Variants Associated with Immune Function in NMOSD patients

Since NMOSD involves an autoimmune component, we focused on variants specifically involved in immune function. Several variant candidates were identified in immune genes (69), including NOTCH1, SPINK5, GUSB, IL6ST, CCR4 and FN1 (fibronectin 1). These genes were all previously documented with deleterious effects of loss of function in heterozygotes, or dose-dependent effects in other disease processes (**Table 2**).

**Table 2:** Selected candidate inherited mutations for NMOSD.

Gene	Chr	Pos	Ref	Alt	func	revel	cadd	gnomadAF	Samples	Other disorders
CCR4	3	32995947	T	C	Dmis	0.374	25.8	3.23E-05	NMO-63	Steven Johnson Syndrome
FN1	2	2.16E+08	G	A	Dmis	0.287	24.6	0.0002	NMO-38; NMO-54	glomerulopathy and spondylometaphyseal dysplasia
FN1	2	2.16E+08	A	G	Dmis	0.252	26.5	3.23E-05	NMO-62	glomerulopathy and spondylometaphyseal dysplasia
FN1	2	2.16E+08	G	A	Dmis	0.448	34	0.0002	NMO-38; NMO-54	glomerulopathy and spondylometaphyseal dysplasia
GUSB	7	65439334	A	T	Dmis	0.914	25.6	0	NMO-31	mucopolysaccharidosis type VII
IL6ST	5	55259272	T	C	Dmis	0.153	22.5	0.0002	NMO-66	arthritis-like autoimmune disease
IL6ST	5	55243391	C	T	Dmis	0.201	33	0	NMO-37	arthritis-like autoimmune disease
NOTCH1	9	1.39E+08	C	T	Dmis	0.169	24.5	0.0004	NMO-27	aortic valve disease, Adams-Oliver syndrome, T-cell acute lymphoblastic leukemia, chronic lymphocytic leukemia, and head and neck squamous cell carcinoma.
NOTCH1	9	1.39E+08	G	A	Dmis	0.325	24.6	6.47E-05	NMO-55	aortic valve disease, Adams-Oliver syndrome, T-cell acute lymphoblastic leukemia, chronic lymphocytic leukemia, and head and neck squamous cell carcinoma.
SPINK5	5	1.47E+08	G	T	Dmis	0.197	24	0	NMO-5	Netherton syndrome

A recent study has indicated that mutations in the complement cascade, specifically complement 4A, can increase genetic susceptibility to NMOSD (47). We observed a marginally significant enrichment of rare damaging missense mutations (MAF<1e-3, REVEL>0.5, Enrichment=3.6, p-value=0.054, binomial test) in the complement pathway. We identified two predicted pathogenic variants, one in C7 and one in C8B, in addition to multiple variants predicted deleterious to protein structure and function by *in silico* algorithms (**Table 3**). A variant documented to cause C8B deficiency, type II was also detected (70, 71). Two variants were detected in C1S, which is associated with Ehlers-Danlos Syndrome, Periodontal Type, 2 and Complement Component C1s Deficiency. The C7, C8B and C9 complement genes are all downstream of the complement C4A gene, which has previously been described as a potential risk factor for NMOSD (47). We did not identify C4A mutations in our NMOSD patient cohort.

**Table 3:** List of damaging rare genetic variants identified by exome sequencing in the genes encoding proteins that are part of the complement system.

Gene	Chr	Pos	Ref	Alt	func	revel	cadd	gnomadAF	Samples
C1S	12	7169873	A	G	Dmis	0.038	22.4	0.0006	NMO-32
C1S	12	7174371	C	T	Dmis	0.151	24.6	0	NMO-31
C3	19	6709721	T	C	Dmis	0.181	25.6	3.24E-05	NMO-69
C7	5	40931185	T	G	Dmis	0.554	26.6	0	NMO-41
C8B	1	57420448	G	C	Dmis	0.868	27.4	3.23E-05	NMO-42
C9	5	39341348	C	T	Dmis	0.579	34	0.0002	NMO-69

### *De novo* Mutations in NMOSD patients

**Figure 2:**
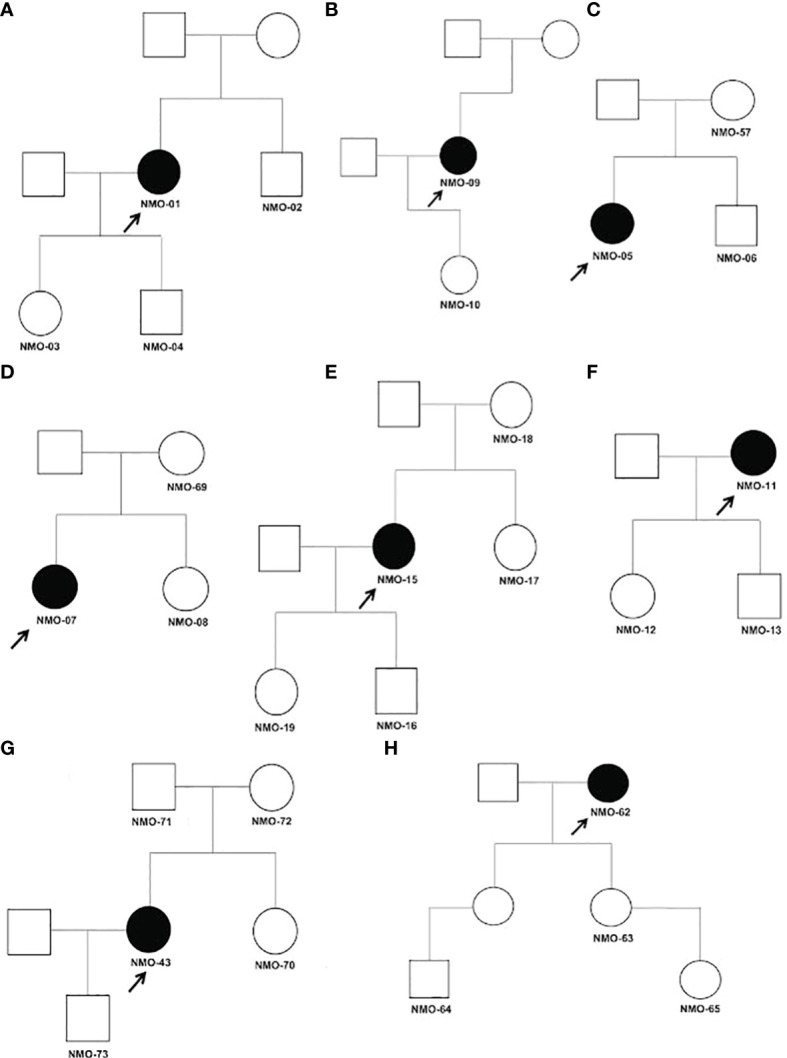
**(A–H)** Pedigrees of tested relatives and probands for trio analysis. Affected individuals are shaded black and probands are indicated by arrows. All individuals tested in our analysis are indicated by their assigned number. Males are represented by squares and females by circles. Detailed clinical data for each family described in **Supplementary Table 4**.

While it is possible for multiple cases of NMOSD to occur within a single family, the disease usually arises in a single family member. It is therefore possible that the disorder arises from multigenic factors, including *de novo* mutations that contribute to disease susceptibility. We identified three *de novo* mutations in patients with NMOSD by whole exome sequencing of trios, including the proband and the proband’s mother and father (**Table 4**). Pedigrees of individuals in this study are illustrated in **Figure 2**.

**Table 4:** *De novo* variants identified in patients with NMOSD.

Proband	Chr	Pos	Ref	Alt	Gene	GeneName	VarType	AAchange	gnomAD_genome_MAF	MetaSVM	CADD	pLI
**NMOSD-31**	3	1.25E+08	G	T	SNX4	sorting nexin 4	missense	SNX4:NM_003794:exon13:c.C1251A:p.D417E	0	T	21	0.01
**NMOSD-43**	4	4E+07	G	A	PDS5A	PDS5 cohesin associated factor A	missense	PDS5A:NM_001100399:exon25:c.C2935T:p.R979C	0	T	34	1
**NMOSD-61**	12	5.2E+07	C	T	BIN2	bridging integrator 2	missense	BIN2:NM_001290008:exon10:c.G1549A:p.E517K-BIN2:NM_001290009:exon10:c.G1177A:p.E393K-BIN2:NM_001290007:exon11:c.G1471A:p.E491K-BIN2:NM_016293:exon11:c.G1645A:p.E549K	0.0003	D	17.79	0

In patient NMO-51, we detected a c.1645G>A (p.Glu549Lys) missense mutation in gene BIN2 (Bridging integrator2). BIN2 is a membrane remodeling adaptor for SH3 domain containing proteins located at dynamic actin-remodeling adhesive structures of immune cells (72). It promotes cell motility and migration, probably *via* its interaction with the cell membrane and with podosome proteins that mediate interaction with the cytoskeleton. In patient NMO-32, we observed a missense c.1251C>A (p.Asp417Glu) mutation in Sorting Nexin 4 (SNX4) gene. SNX4 has been associated with Alzheimer’s disease (AD) (73). SNX4 regulates β-site amyloid precursor protein-cleaving enzyme 1 (BACE1) trafficking, overexpression of SNX4 significantly increased the levels of BACE1 and β-amyloid (Aβ) in AD. In patient NMO-43, a missense mutation c.2935C>T (p.Arg979Cys) in the *PDS5A* gene, the protein encoded by this gene binds to the cohesin complex and associates with chromatin through most of the cell cycle. The encoded protein may play a role in regulating sister chromatid cohesion during mitosis.

### Common variants associations

**Figure 3:**
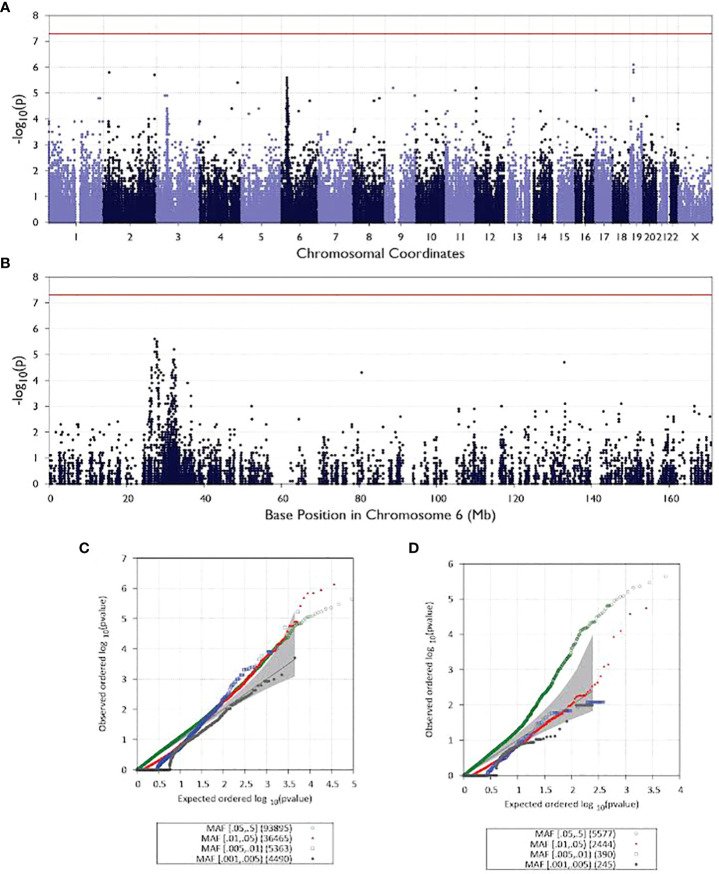
Manhattan and QQ plots for all analyzed patients. All chromosomes are illustrated as Manhattan plots in **(A)** and QQ plots in **(C)**. Manhattan plot for chromosome 6 in **(B)** and QQ plots for the same chromosome in **(D)**.

In addition to analyzing rare and *de novo* mutations in patients, we also tested 200,292 common variants that were found in our exome sequencing dataset for associations with NMOSD. A Manhattan plot is shown in **Figure 3A** and **Figure 3B** shows the GWAS signal on chromosome 6. While there is a peak associated with the variant, it does not rise to the level of statistical significance, which is likely due to the small sample size of our study. **Figure 3C** shows a a QQ plot of the test results. While multiple common variants were observed to be more prevalent in NMOSD patients than in the general population, none of them reached statistical significance.

We further tested whether our population sample could validate the results of a recent study (47) of NMOSD. The previous study used GWAS that identified 2 common SNPs as associated with NMOSD. These SNPs are rs28383224 (MAF = 0.42, odds ratio = 2.24, p-value = 5.8 × 10−12) mapped 21.5 kb downstream of HLADQA1; and rs1150757 (MAF = 0.10, odds ratio = 4.66, p-value = 3.33 × 10−16). Our analysis included only one of the previously identified SNPs, as rs28383224 occurs outside the coding sequence and is therefore not picked up by WES. The second variant identified in the previous study, rs1150757, was also observed in our dataset (MAF = 0.096 in controls and 0.25 in cases, p-value = 1.52 x 10^-5^).

### HLA typing of Serbian Patient Population

Specific HLA alleles are associated with certain autoimmune diseases, and several previous studies were able to observe an association between HLA alleles and NMOSD. However, studies in other populations did not observe this association. One potential explanation for this result is the possibility of linkage disequlibrium with HLA alleles and NMOSD risk loci. To address this possibility, we analyzed HLA haplotypes in NMOSD and compared the prevalence of these haplotypes with the general population. We were able to identify a haplotype association with NMOSD in our Serbian patients. Frequencies of HLA haplotypes, including HLA-A, -B, -DRB1 and HLA-A, -B, -C, -DRB1, -DQB1, have previously been reported in this population (52, 53). We were then able to use these previously available data to calculate the estimated corresponding haplotype frequencies for comparison to the patient population and family members (full comparison result is in **Supplementary Table 4****)**. Detailed clinical data for these patients is in **Supplementary Table 4**. The most significant difference we observed between the Serbian population and the NMOSD patients is in the haplotypes HLA-A*01, B*08, DRB1*03 and HLA-A*01, B*08, C*07, DRB1*03, DQB1*02. We observed an increased prevalence of the haplotype HLA-A*01, B*08, DRB1*03, at a frequency of 0.21154 in patients, as opposed to 0.059114 in the Serbian population overall (p-value = 2e-4, Fisher exact test) (**Supplementary Table 5**, Serbia_pop3). Likewise, there was an increased prevalence of haplotype HLA-A*01, B*08, C*07, DRB1*03, DQB1*02, with a frequency of 0.21154 in NMOSD patients, as opposed to 0.066038 in Serbia_pop3 (p-value = 5 x 10^-4^, Fisher exact test).

Multiple analysis of Serbian haplotypes are available, and a comparison between them indicates that the haplotype frequencies used for our analysis are accurate. As shown in **Supplementary Table 5**, the HLA allele frequencies are similar between two datasets we could have used for our analysis, Serbia_pop3 and Serbia_Vojvodina. A short list of the HLA alleles with more than 0.03 (3%) difference in frequency between our patients and Serbia_pop3 is in **Tables 5** and **6**.

**Table 5:** HLA alleles with more than 0.03 (3%) difference in allele frequencies between the patients and Serbia_pop3.

Allele	% of patients having the allele	% of Serbians having the allele (Serbia_pop3)	Difference in % of individuals	Allele frequency in patients	Allele frequency in Serbia_pop3	Difference in allele frequency	Pvalue (fisher_exact_test)
DRB1*03	57.69	21	36.69	0.2885	0.1087	0.1798	0.000345819
B*08	42.31	16.47	25.84	0.2115	0.0864	0.1251	0.021468107
DQB1*02	57.69	30.2	27.49	0.2885	0.1698	0.1187	0.038757941
DQB1*05	73.08	54.7	18.38	0.4423	0.3302	0.1121	0.18154181
A*01	46.15	26.25	19.9	0.25	0.1426	0.1074	0.04339775
DRB1*14	23.08	10.7	12.38	0.1154	0.0542	0.0612	0.062586003
DRB1*01	23.08	19.4	3.68	0.1538	0.1029	0.0509	0.486565697
DRB1*08	11.54	6.2	5.34	0.0769	0.0309	0.046	0.220410998
DRB1*16	30.77	20.4	10.37	0.1538	0.1087	0.0451	0.502538467
C*07	46.15	44.02	2.13	0.2885	0.2484	0.0401	0.518981233
C*12	30.77	25.79	4.98	0.1731	0.1352	0.0379	0.414094602
C*03	19.23	11.95	7.28	0.0962	0.0598	0.0364	0.240012755
DQB1*04	11.54	4.4	7.14	0.0577	0.022	0.0357	0.108662136
A*33	11.54	4.92	6.62	0.0577	0.0254	0.0323	0.149070771
B*13	0	6.53	-6.53	0	0.0329	-0.0329	0.415394241
A*24	15.38	21.18	-5.8	0.0769	0.1109	-0.034	0.367454104
A*03	15.38	21.13	-5.75	0.0769	0.1132	-0.0363	0.270804152
B*51	15.38	23.24	-7.86	0.0769	0.1285	-0.0516	0.14625227
DRB1*15	7.69	18.6	-10.91	0.0385	0.0984	-0.0599	0.233720459
C*06	7.69	18.87	-11.18	0.0385	0.1006	-0.0621	0.16514706
DQB1*06	15.38	27	-11.62	0.0769	0.1447	-0.0678	0.107143485
DRB1*07	0	13.9	-13.9	0	0.0713	-0.0713	0.049110681
B*44	3.85	17.22	-13.37	0.0192	0.0916	-0.0724	0.012729771
DRB1*11	19.23	31	-11.77	0.0962	0.1689	-0.0727	0.193298849
DRB1*13	7.69	24.9	-17.21	0.0577	0.1323	-0.0746	0.146018754
DRB1*04	0	17	-17	0	0.0899	-0.0899	0.012695386
DQB1*03	26.92	54.7	-27.78	0.1346	0.3333	-0.1987	0.000522299

The table is sorted by the difference in allele frequency (last column). The full list is in the supplementary file 1 data sheet “Belgrade_HLA.”

Bold values indicate significant alleles.

“*” means by DNA sequencing.

**Table 6:** Top predicted HLA class II binding peptides in AQP4 and MOG.

Protein	Position	Peptide	Restricted HLA allele
Aquaporin-4	29-48	VWTQAFWKAVTAEFLAMLIF	DQA1*0501-DQB1*0201, A*0101, and C*0701
Aquaporin-4	246-263	GLYEYVFCPDVEFKRRFK	DRB1*0301, DQA1*0501-DQB1*020, and C*0701
MOG	38-57	RHPIRALVGDEVELPCRISP	DRB1*0301 and DQA1*0501-DQB1*0201
MOG	233-247	RRLAGQFLEELRNPF	DRB1*0301, DQA1*0501-DQB1*0201, A*0101, and B*0801

“*” means by DNA sequencing.

### Molecules encoded by HLA alleles enriched in NMOSD patients can bind disease-relevant antigens

We performed *in silico* modeling of the binding of proteins encoded by these two haplotypes to two NMOSD-relevant antigens, AQP4 and MOG.

The *in sillico* modeling generated binding predictions for both HLA class I and class II molecules in the haplotype we identified to AQP4 (**Supplementary Tables 7** and **8**) and MOG (**Supplementary Tables 9** and **10**). We first examined the haplotype HLA-A*01, B*08, C*07, DRB1*03, DQB1*02. We observed that there were two class I molecules that were able to bind peptides within AQP4. The 9-mer peptide at position 104, MVCTRKISI, was predicted to bind B*0801 and C*0701 (**Supplementary Table 7**). This 9-mer is part of the 15-mer peptide, MVCTRKISIAKSVFY, that is predicted to bind DRB1*0301 (**Supplementary Table 8**).

Our *in silico* modeling of class II binding to AQP4 (74) generated multiple predicted binding peptides, which are summarized in **Supplementary Table 14** and **Supplementary Table 15**. In AQP4, the 20-mer peptide, VWTQAFWKAVTAEFLAMLIF, is predicted to bind DQA1*0501-DQB1*0201 (**Supplementary Table 8**) and contains a weaker A*0101 binder, WTQAFWKAV, and a weaker C*0701 binder, VTAEFLAML (**Supplementary Table 7**). The 18-mer peptide, GLYEYVFCPDVEFKRRFK, is predicted to bind DRB1*0301, DQA1*0501-DQB1*0201 (**Supplementary Table 8**) and contains a weaker C*0701 binder, YEYVFCPDV (**Supplementary Table 7**). In MOG, the 15-mer peptide, RRLAGQFLEELRNPF, is predicted to bind DRB1*0301 and DQA1*0501-DQB1*0201 (**Supplementary Table 7**) and contains a promiscuous weak 9-mer binder, FLEELRNPF, predicted to bind A*0101 and B*0801 (**Supplementary Table 9**).

**Figure 4:**
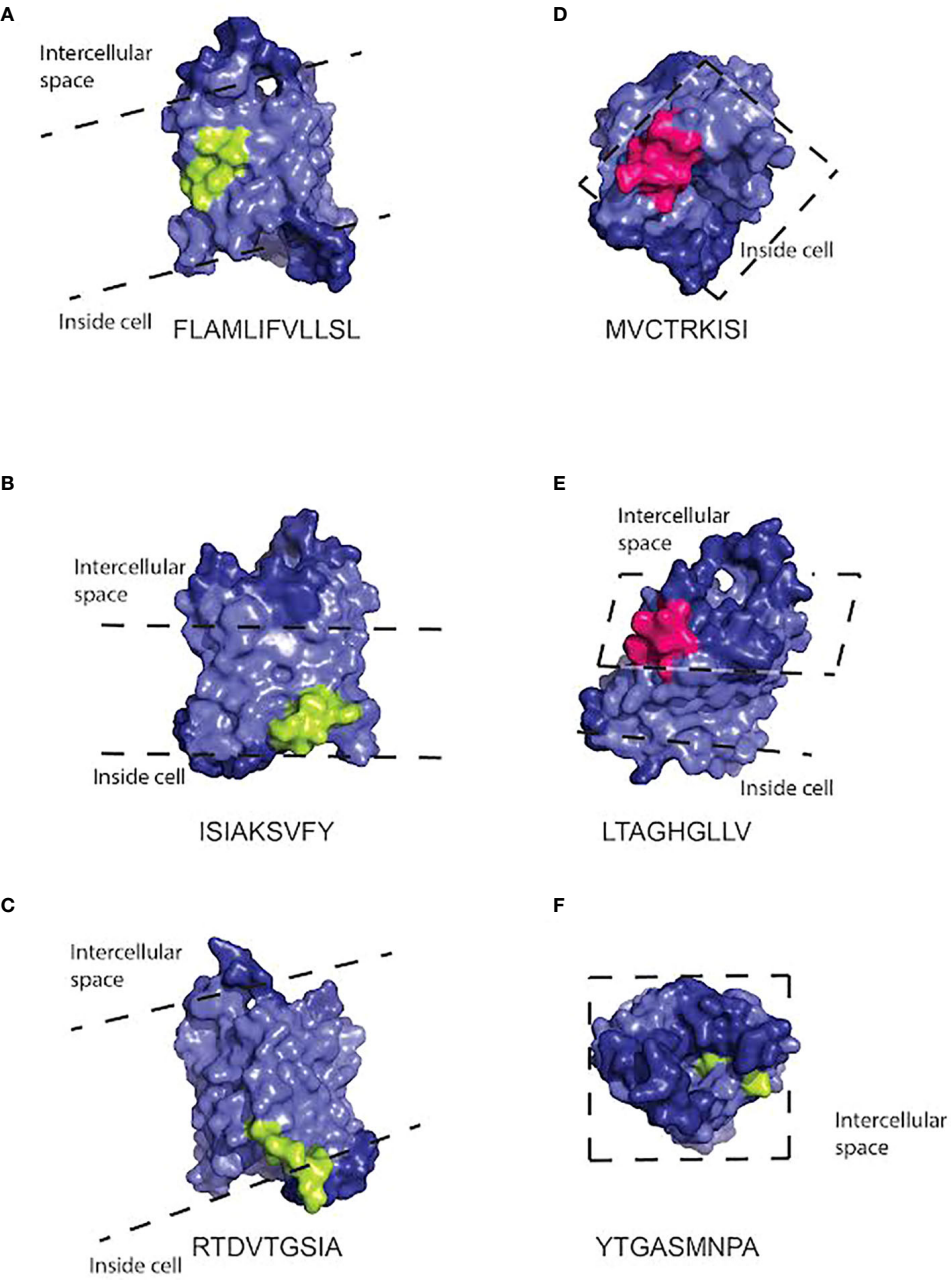
Three dimensional space-filling cartoon of the AQP4 molecule positioned so that peptides capable of binding HLA class I molecules in the haplotype (either A*0101, B*0801, or C*0701) are visible. The pale blue section of the molecule indicates the intramembrane portion. Dashed lines indicate the approximate position of the extracellular and intracellular surfaces of the cell membrane. Peptides that bind HLA class I molecules strongly are indicated in light green. Promiscuous binders are indicated in pink. Peptide sequences and the HLA molecules binding to them are listed under each model. Peptide sequences: **(A)** FLAMLIFVLLSL **(B)** ISIAKSVFY **(C)** RTDVTGSIA **(D)** MVCTRKISI **(E)** LTAGHGLLV **(F)** YTGASMNPA.

**Figure 5:**
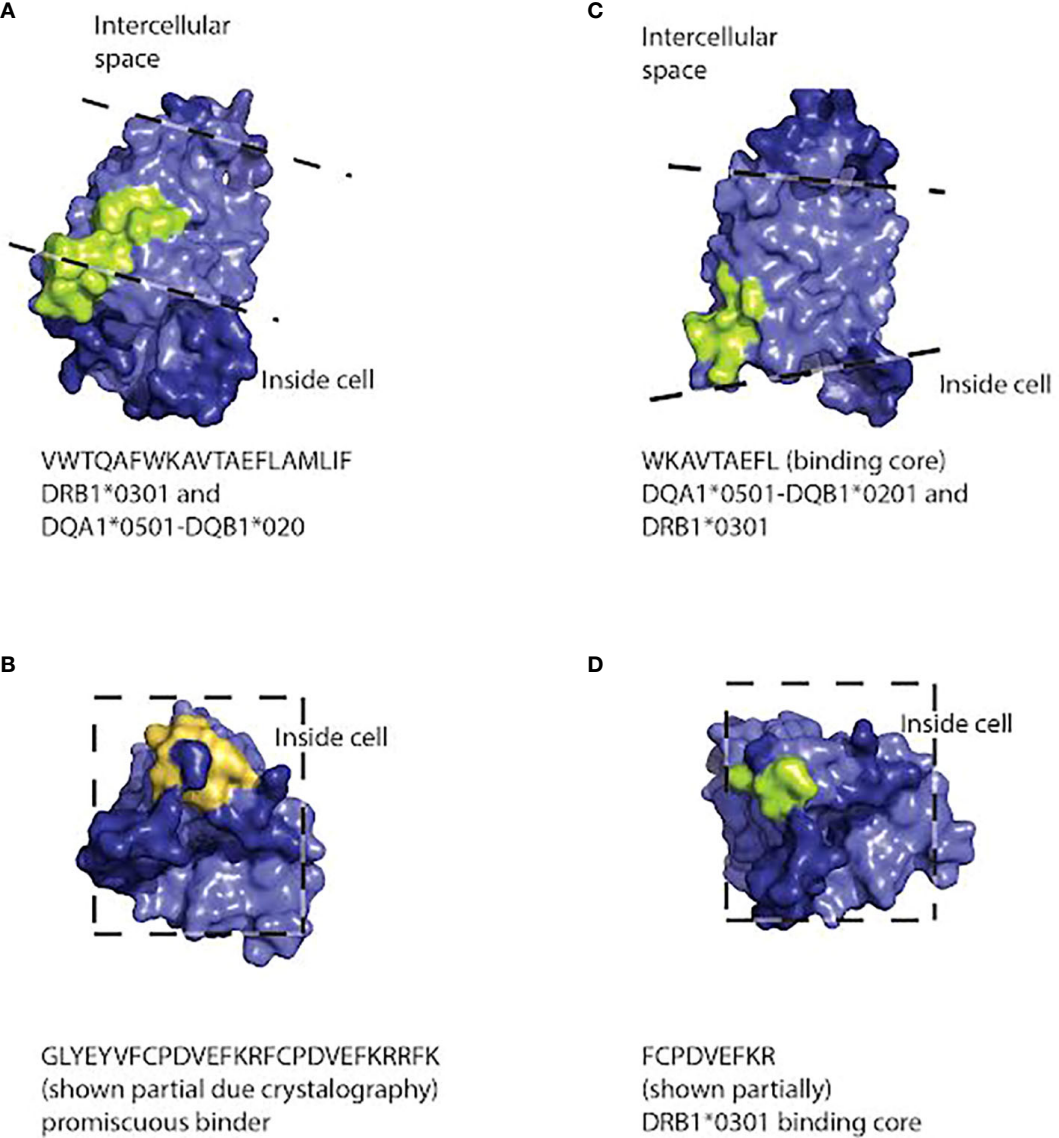
Three dimensional space-filling diagram of the AQP4 molecule positioned so that peptides capable of binding HLA class II molecules (DRB1*0301, DQA1*0501-DQB1*0201) are visible. The pale blue section of the molecule indicates the intramembrane portion. Dashed lines indicate the approximate position of the extracellular and intracellular surfaces of the cell membrane. Peptides that bind HLA class II molecules strongly are indicated in light green. Promiscuous binders are indicated in orange. Peptide sequences and the HLA molecules binding to them are listed under each model. Peptide sequences: **(A)** VWTQAFWKAVTAEFLAMLIF binding to DRB1*0301 and DQA1*0501-DQB1*020. **(B)** GLYEYVFCPDVEFKRFCPDVEFKRRFK binds promiscuously **(C)** WKAVTAEFL binding DQA1*0501-DQB1*0201 and DRB1*0301 **(D)** FCPDVEFKR binds DRB1*0301.

To generate models of which cellular locations and functional domains of the AQP4 molecule may be targeted by the class I and class II molecules, we used the structure of the protein based on X-ray crystallography (74) to predict the location of the binding sites to human AQP4 in PyMol (**Figures 3**, **4**). The binding sites of HLA class I alleles were observed to occur within, on the inside and on the outside of the cell membrane, primarily on transmembrane domains, within the portion of the AQP4 molecule where a crystal structure was available. For HLA class II, the binding sites occurred inside the membrane or inside the cell, for the portion of the AQP4 molecule where a structure was available (**Figure 5**)

## Discussion

### HLA Typing

Previous studies from multiple populations have shown an association between HLA DRB1*0301 and NMOSD, but studies in other populations have not (38). Our results are consistent with the recent meta-analysis that indicated that HLA DRB1*0301 is associated with NMOSD in populations from Europe and North America, but not populations from Asian countries (38). One potential explanation for different associations of NMOSD and HLA alleles in different populations is that an allele that is inherited in the same haplotype block as DRB1*03 actually confers NMOSD susceptibility in some populations, but that in other populations, this allele is part of a different haplotype block. Exploring this hypothesis further requires not just looking at DRB1*03, but rather analyzing the haplotype block of which it is a part. Our analysis of Serbian NMOSD patients indicated that the haplotypes HLA-A*01, B*08, DRB1*03 and HLA-A*01, B*08, C*07, DRB1*03, DQB1*02 are more common in affected individuals than in the Serbian population. Our results indicate that DRB1*03 is associated with a particular allele configuration at this locus, and therefore, a number of alleles in this haplotype block are strong candidates for conferring susceptibility to NMOSD in the Caucasian Serbian population. It is possible that only certain alleles in this haplotype increase NMOSD susceptibility. Alternatively, the alleles present within this haplotype block could modify each other, in which case only a particular configuration of alleles would increase NMOSD susceptibility. Future sequencing experiments in multiple populations would help distinguish between these possibilities, as would functional and mechanistic studies.

Regarding systems for predicting HLA binding, Gowthaman et al. (2010) evaluated eight prediction systems in predicting peptides binding to seven HLA class I alleles, A*0101, 0201, 0301, 1101, 2402, B*0702, and 0801 and found that NetMHC, NetMHCpan, and IEDB showed high overall accuracy (75). Lin et al. evaluated 21 HLA class II prediction methods for their performance in predicting binding peptides of seven common HLA-DR allele, DRB1*0101, 0301, 0401, 0701, 1101, 1301, and 1501 (76) and found that the HLA class II predictors do not match prediction capabilities of HLA class I predictors. It is more difficult to predict MHC class II binding as, unlike MHC class I molecules, can accommodate the 9-mer binding core of the peptides inside while peptide termini protrude outside of the grooves (77). NetMHCII is one of the top predictors for DRB1*0301, and was therefore used in our study. We used different predictors for class I and class II binding, to maximize accuracy.

To test a potential mechanism by which that haplotype could confer susceptibility, we predicted the likely binding sites of the HLA alleles to both AQP4 and MOG because both have been shown to be involved in NMOSD.

The illustrations of our peptide predictions used a published crystal structure of AQP4, 3GD8 (74). This crystal structure has a 1.8 angstrom resolution and it includes all the transmembrane domains, but it doesn’t include the N- and C- terminals of the protein, most likely because their structure is less stable. Thus, the peptides on the C-terminus, including the PDZ domain, were not fully illustrated on the crystal structure. For the incomplete crystal structure available to us, it was notable that most of the peptides predicted to bind HLA alleles are located on the intracellular surface of the molecule or, in one case, inside the pore. Immune cells are unlikely to encounter these peptides unless they are processed and presented on the surface of the cell, but if that does occur, the combination of the peptides and HLA alleles capable of binding them could trigger NMOSD. Since NMOSD are frequently characterized by anti-AQP4 antibodies, it is likely that there is a way that these peptides would be presented on the cell surface. For example, complement-dependent cytotoxicity is thought to be a major mechanism of NMOSD (78). It may also allow intracellular domains of AQP4 to interact with additional immune cells.

Our results are consistent with other epitope mapping studies of AQP4. Based on our current analysis, we have proposed several distinct antigens of AQP4 as predicted based on binding specificities in HLA class I and HLA class II molecules. Future studies to investigate the T cell responses in NMOSD patient PBMCs to these predicted antigens are necessary for discovery and validation of autoimmune NMOSD epitopes. Determining the correct autoimmune antigens may aid in the development of targeted therapies in these patients.

### Whole exome sequencing

Many genes idenfiied by WES are also implicated in other disorders. Mutations in the NOTCH1 gene are associated with aortic valve disease, Adams-Oliver syndrome, T-cell acute lymphoblastic leukemia, chronic lymphocytic leukemia, and head and neck squamous cell carcinoma (79–81). At least some of the mutations thought to be responsible for Adams-Oliver syndrome in patients are heterozygous (82). SPINK5 (serine peptidase inhibitor, Kazal type 5) are associated with Netherton syndrome (OMIM#256500), a disorder involving skin and hair abnormalities and a high risk of allergies, asthma, and an inflammatory skin condition, eczema. Homozygous loss of function in GUSB causes mucopolysaccharidosis type VII (also known as Sly disease) (83, 84), which is characterized by accumulation of glycosaminoglycans within the lysosomes, leading to severe defects in the structure of tissues and organs. Mutations of IL6ST in mice can result in a hyper-responsive T cell phenotype, and development of an arthritis-like autoimmune disease in mice (85). Clinical trials of the anti-CCR4 antibody Mogamulizumab revealed the depletion of Treg cells in a subset of patients, coupled with extreme infiltration of T cells into the skin, resulting in Steven Johnson Syndrome (OMIM#608579) (86, 87). An *in vitro* model of bladder cancer, a partial decrease in the expression of CCR4 has been shown to facilitate metastasis, despite residual protein activity. Certain heterozygous mutations in FN1 have been proposed to cause or contribute to disease, including glomerulopathy and spondylometaphyseal dysplasia (88, 89).

These genes also have important function in the central-nervous system and immune systems. IL6ST (interleukin 6 signal transducer, also known as GP130) activate another candidate gene NOTCH1 in mouse and human cells (90) and has been implicated in the survival of motor and sensory neuron subtypes in mice (91). CCR4 (C-C Chemokine Receptor 4) is expressed on CD4+ thymocytes, and is potentially involved in negative selection in the thymus (92). CCR4 also expressed on multiple types of T cells, including Th2, Th17, Th22, and Treg (86). FN1 is a component of the blood brain barrier, which is also expressed in astrocytes (93). In rats, it plays a critical role in the axonal regeneration of adult white matter after injury, at least in culture (93). All three genes (SNX4, PDS5A and BIN2) identified by *de novo* mutations are highly specifically expressed in EBV-transformed lymphocytes.

While prior research shows that NMOSD likely has some genetic associations, it is rare to identify multiple cases of the disease within a single family, suggesting that disease susceptibility may be a result of a variety of factors. Recent studies have shown that *de novo* variation in the coding sequences of several genes confer disease susceptibility in probands with neurodevelopmental and psychiatric disorders from otherwise healthy families (50, 51, 94). The results of our trio analysis are consistent with this possibility, as the analysis revealed multiple *de novo* mutations, including predicted pathogenic variants associated with immune function. It is therefore possible that some of these alleles contribute to the development of NMOSD in the probands in this study.

We found that certain alleles from several genes were more prevalent in patients with NMOSD than in controls represented by the gnomAD, indicating that the variants identified in our cohort of patients with NMOSD are presumed to be rare. Certain among these alleles, such as the complement alleles were involved in pathways that have previously been proposed to contribute to the genetic risk of NMOSD (95). While we did not identify the same complement mutation (C4A) as a previous study on NMOSD, we identified mutations in a variety of molecules that are downstream of C4A. The likely explanation for this difference is that our study population of NMOSD patients originated from a different region, and thus, different pathogenic polymorphisms would be expected to be present in this population. The identification of variants in complement genes, including pathogenic mutations, in the NMOSD patient population supports a role for the complement cascade in the pathogenesis of this disease. None of the variants we identified overlapped with the variants detected in the re-analysis of the Estrada et al. data (47, 48), or of a separate association study on a group of Japanese patients (49). However, the latter study identified a variant in the gene encoding a voltage gated potassium channel, KCNMA1, as increasing susceptibility to NMOSD (49). While we did not see a relationship between this particular gene and NMOSD susceptibility in our data, we identified a variety of potentially deleterious rare variant mutations in NMOSD patients, and potassium channels as a category were detected by Enrichr (**Supplementary Tables 1**, **2**). Given the well-documented role of astrocytes in buffering potassium ions to maintain neuronal membrane potentials, ability to signal and viability, and the involvement of AQP4 in this process, it is reasonable that otherwise benign variations in potassium channels may contribute to the progression of NMOSD. Further analysis of patient populations combined with targeted genetic testing in disease models, including functional studies using patient-specific cells, are necessary to further elucidate the significance of these variants with the ultimate goal to design targeted therapies that reduce progression of NMOSD.

## Coinvestigators

# Regeneron Genetics Center Banner Author List

All authors/contributors are listed in alphabetical order.

RGC Management and Leadership Team

Goncalo Abecasis, Aris Baras, Michael Cantor, Giovanni Coppola, Andrew Deubler, Aris Economides, Luca A. Lotta, John D. Overton, Jeffrey G. Reid, Alan Shuldiner, Katia Karalis and Katherine Siminovitch

Sequencing and Lab Operations

Christina Beechert, Caitlin Forsythe, Erin D. Fuller, Zhenhua Gu, Michael Lattari, Alexander Lopez, John D. Overton, Thomas D. Schleicher, Maria Sotiropoulos Padilla, Louis Widom, Sarah E. Wolf, Manasi Pradhan, Kia Manoochehri, Ricardo H. Ulloa.

Genome Informatics

Xiaodong Bai, Suganthi Balasubramanian, Boris Boutkov, Gisu Eom, Lukas Habegger, Alicia Hawes, Shareef Khalid, Olga Krasheninina, Rouel Lanche, Adam J. Mansfield, Evan K. Maxwell, Mona Nafde, Sean O’Keeffe, Max Orelus, Razvan Panea, Tommy Polanco, Ayesha Rasool, Jeffrey G. Reid, William Salerno, Jeffrey C. Staples,

Research Program Management

Marcus B. Jones and Lyndon J. Mitnaul

## Data availability statement

The datasets presented in this study can be found in online repositories. The name of the repository and accession number can be found below: NCBI database of Genotypes and Phenotypes (dbGaP); accession phs003055.v1.p1.

## Ethics statement

The studies involving human participants were reviewed and approved by Northwell Health Institutional Review Board. The patients/participants provided their written informed consent to participate in this study.

## Author contributions

IT, AT, and JW designed experiments, interpreted/analyzed data, performed experiments and wrote the paper. GZ performed HLA sequencing analysis and wrote the paper. ID and SM provided expertise on NMOSD, patient samples, and wrote the paper. PJ designed and ran HLA sequencing experiments. DK designed HLA sequencing experiments, interpreted data and wrote the paper. SN provided neurobiology expertise and support for the project, and interpreted data. JNHS designed and supervised experiments, interpreted data, and wrote the paper. VB helped write paper. YS oversaw bioinformatics analysis on exome sequencing. AK, JD, EY, VJ, TdA, MF, AH, MM, MS, VN, WdG, ML, KB, MG provided clinical expertise, recruited patients and processed patient samples.

Regeneron Genetics Center Banner Author List and Contribution Statements

All authors/contributors are listed in alphabetical order.

RGC Management and Leadership Team

Goncalo Abecasis, Aris Baras, Michael Cantor, Giovanni Coppola, Andrew Deubler, Aris Economides, Luca A. Lotta, John D. Overton, Jeffrey G. Reid, Alan Shuldiner, Katia Karalis and Katherine Siminovitch

Contribution: All authors contributed to securing funding, study design and oversight. All authors reviewed the final version of the manuscript.

Sequencing and Lab Operations

Christina Beechert, Caitlin Forsythe, Erin D. Fuller, Zhenhua Gu, Michael Lattari, Alexander Lopez, John D. Overton, Thomas D. Schleicher, Maria Sotiropoulos Padilla, Louis Widom, Sarah E. Wolf, Manasi Pradhan, Kia Manoochehri, Ricardo H. Ulloa.

Contribution: CB, CF, AL, and JO performed and are responsible for sample genotyping. CB, CF, EF, ML, MP, LW, SW, AL, and JO performed and are responsible for exome sequencing. TS, ZG, AL, and JO conceived and are responsible for laboratory automation. MP, KM, RU, and JO are responsible for sample tracking and the library information management system.

Genome Informatics

Xiaodong Bai, Suganthi Balasubramanian, Boris Boutkov, Gisu Eom, Lukas Habegger, Alicia Hawes, Shareef Khalid, Olga Krasheninina, Rouel Lanche, Adam J. Mansfield, Evan K. Maxwell, Mona Nafde, Sean O’Keeffe, Max Orelus, Razvan Panea, Tommy Polanco, Ayesha Rasool, Jeffrey G. Reid, William Salerno, Jeffrey C. Staples,

Contribution: XB, AH, OK, AM, SO, RP, TP, AR, WS, and JR performed and are responsible for the compute logistics, analysis and infrastructure needed to produce exome and genotype data. GE, MO, MN, and JR provided compute infrastructure development and operational support. SB, SK, and JR provide variant and gene annotations and their functional interpretation of variants. EM, JS, RL, BB, AB, LH, and JR conceived and are responsible for creating, developing, and deploying analysis platforms and computational methods for analyzing genomic data.

Research Program Management

Marcus B. Jones and Lyndon J. Mitnaul

Contribution: All authors contributed to the management and coordination of all research activities, planning and execution. All authors contributed to the review process for the final version of the manuscript.

## Funding

IT was supported by a NICHD F32 081835 and a Kavli Institute Pilot Grant. IT, VN, MF, WD, and JS were supported by a grant from the HF Langbert Neuroimmunology Research Fund. MF was funded in part by the Cheryl Manne Research Fellowship.

## Acknowledgments

We are very grateful to Dr Wendy Chung for reading our manuscript and providing essential feedback for our study.

## Conflict of interest

The members of the Regeneron Genetics Center and AK were employed by Regeneron.

The remaining authors declare that the research was conducted in the absence of any commercial or financial relationships that could be construed as a potential conflict of interest.

## Publisher’s note

All claims expressed in this article are solely those of the authors and do not necessarily represent those of their affiliated organizations, or those of the publisher, the editors and the reviewers. Any product that may be evaluated in this article, or claim that may be made by its manufacturer, is not guaranteed or endorsed by the publisher.

## Supplementary material

Supplementary Table 1 346 variants in 338 genes that identified by PSAP model.

Supplementary Table 2 Reactome analysis of missense variants identified in the dataset.

Supplementary Table 3 List of selected rare variants identified by exome sequencing in genes involved in immune function. Full list of detected variants is available in supplementary information.

Supplementary Table 4 Clinical features and identifiers of probands and family members in trio analysis.

Supplementary Table 5 Comparison of estimated haplotype frequencies between the Belgrade population (using previously available data) and the patient population and family members included in the present study.

Supplementary Table 6 Detailed clinical information from Belgrade patients.

Supplementary Table 7 Summary of the predicted HLA class I binding peptides in AQP4. Predicted weak binders, strong binders, and promiscuous binders (peptides predicted to bind multiple HLA alleles) are highlighted in green, yellow and red, respectively.

Supplementary Table 8 Predicted HLA class II binding peptides in AQP4. Predicted weak binders, strong binders, and promiscuous binders (peptides predicted to bind multiple HLA alleles) are highlighted in green, yellow and red, respectively.

Supplementary Table 9 Predicted HLA class I binding peptides in MOG. Predicted weak binders, strong binders, and promiscuous binders (peptides predicted to bind multiple HLA alleles) are highlighted in green, yellow and red, respectively.

Supplementary Table 10 Predicted HLA class II binding peptides in MOG. Predicted weak binders, strong binders, and promiscuous binders (peptides predicted to bind multiple HLA alleles) are highlighted in green, yellow and red, respectively.
